# Increased levels of prostaglandin E-major urinary metabolite (PGE-MUM) in active mesenteric panniculitis patients: A case report

**DOI:** 10.1097/MD.0000000000009237

**Published:** 2017-12-22

**Authors:** Shinta Mizuno, Masatoshi Wakui, Yujiro Machida, Naoki Hosoe, Tadakazu Hisamatsu, Takashi Ishida, Kaori Kameyama, Makoto Naganuma, Takanori Kanai

**Affiliations:** a Division of Gastroenterology and Hepatology, Department of Internal Medicine; b Department of Laboratory Medicine; c Center for Diagnostic and Therapeutic Endoscopy; d The Third Department of Internal Medicine, Kyorin University School of Medicine; e Department of Surgery; f Department of Pathology, Keio University School of Medicine, Tokyo, Japan.

**Keywords:** biomarker, mesenteric panniculitis, prostaglandin E-major urinary metabolite

## Abstract

**Rationale:**

Mesenteric panniculitis (MP) is a rare disease with abdominal and systemic symptoms and is characterized by nonspecific inflammation, fat necrosis, and fibrosis in mesenteric fat. Active inflammatory responses may increase levels of prostaglandin E-major urinary metabolite (PGE-MUM), which was reported to reflect the disease activity of ulcerative colitis and chronic fibrosing interstitial pneumonia. We recently experienced a case with elevated PGE-MUM at the time of diagnosis of MP and we investigated the potential of PGE-MUM as a biomarker.

**Patient concern:**

In this report we described 2 active mesenteric panniculitis patients with high PGE-MUM levels.

**Diagnoses:**

Mesenteric panniculitis

**Interventions:**

Both MP patients were measured the levels of PGE-MUM.

**Outcomes:**

Both MP patients exhibited high levels of PGE-MUM before treatment. In one, the levels were sensitively correlated with clinical symptoms and serological markers on steroids.

**Lessons:**

The study observations suggest the potential of PGE-MUM to reflect the disease activity of MP. To verify its use, more findings based on clinical studies should be accumulated.

## 1 Introduction

Mesenteric panniculitis (MP) first described in 1924 is an uncommon disease characterized by various processes including nonspecific inflammation, fibrosis, necrosis, and adipose degeneration of mesentery.^[1]^ It can be inferred from these pathological findings that the dominant fibroinflammatory lesion was not mucous membrane but submucous to tela subserosa. The pathological findings of MP are sclerosing mesenteritis and retractile mesenteritis,^[2]^ which are synonymous with Pfeiffer–Weber–Christian disease, sclerosing mesenteritis, liposclerotic mesenteritis, retractile mesenteritis, and misty mesentery. Although there are no specific clinical symptoms of MP, abdominal pain, bloating, and diarrhea are common. The common site of MP is the root of the small bowel mesentery. Immunoglobulin (Ig) G4-related sclerosing disease, malignant lymphoma and other malignancies are thought to cause MP.^[3]^ Sharma et al reported a systematic review of 192 MP cases. According to their report, steroid therapy was most commonly used and frequently followed by treatment with colchicine, tamoxifen, and 6-mercaptopurine. The typical finding from computer tomography (CT) is a soft tissue mass in the mesentery. C-reactive protein (CRP) and erythrocyte sedimentation rate were elevated in most cases with active MP.^[4]^ However, there are no specific laboratory findings concerning MP.

Prostaglandin E2 (PGE2) is synthesized from arachidonic acid via a cyclooxygenase (COX)-catalyzed reaction, and is subsequently metabolized to the stable end-product prostaglandin E-major urinary metabolite (PGE-MUM) by 15-hydroxyprostaglandin dehydrogenase.^[5]^ A previous report showed that PGE-MUM was elevated in patients with active ulcerative colitis (UC) and that the cutoff value for histological UC activity and remission was 17.0 μg/g Cre.^[6]^ In patients with chronic fibrosing interstitial pneumonia (CFIP), PGE-MUM levels were also significantly higher than in controls.^[7]^ These results suggest that PGE-MUM is a promising biomarker that might reflect disease activity. This study investigated the potential of PGE-MUM as a biomarker for MP, summarizing 2 clinically suggestive cases.

This article was written according to CARE guidelines and informed consent was obtained.

## 2 Case reports

**Figure 1:**
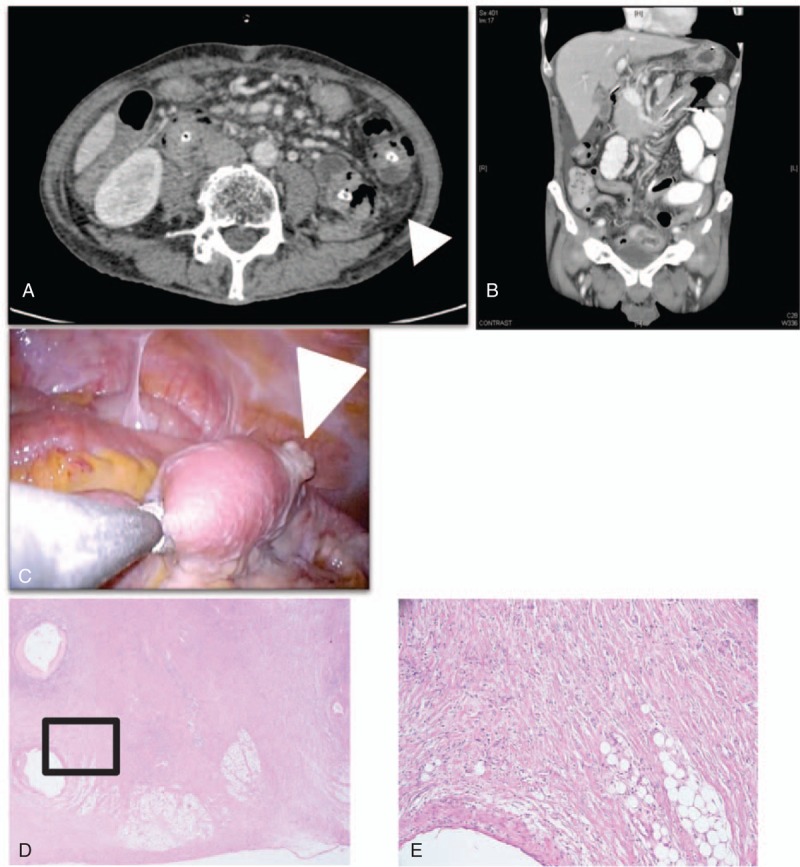
(A) Contrast-enhanced CT performed before operation showed pseudocapsule (arrow head). (B) Coronal CT showed bowel wall thickening. (C) Laparoscopy reveals the protuberating reddish plaque on the ileum (arrow head). (D) Hematoxylin and eosin staining of mesentery showed significant fibrosis, magnification 20×. (E) Higher magnification of the highlighted area (black frame), magnification 100×.

**Figure 2:**
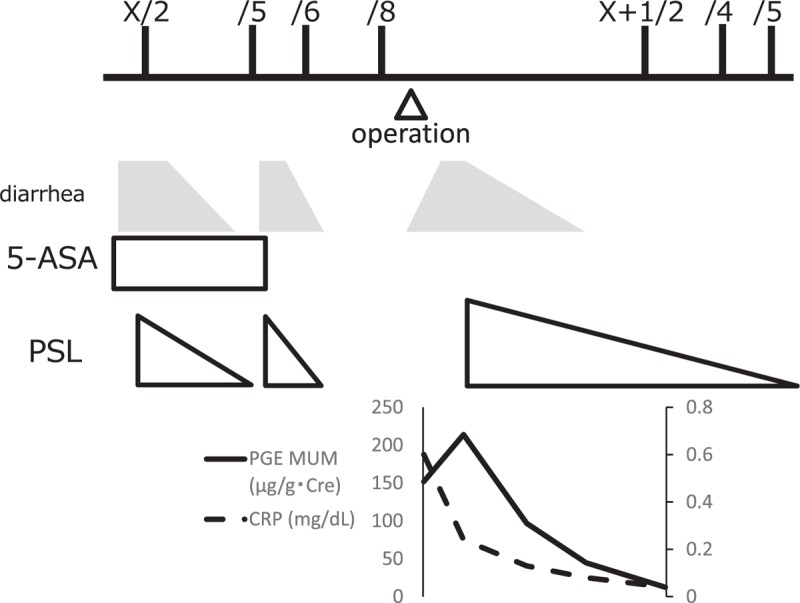
The clinical presentation correlated with PGE-MUM levels after an initiation of steroid therapy.

Case 1: A 64-year-old man with a 2-week history of intermittent diarrhea was previously seen in another hospital. Colonoscopy showed erosive change in the rectum and sigmoid colon, and he was diagnosed with UC. He received 5-aminosalicylate (5-ASA), but his symptoms did not resolve regardless of 5-ASA treatment. He was prescribed additional treatment with prednisolone and immediately responded to that therapy. After the discontinuation of steroid therapy, intermittent diarrhea relapsed in 2 weeks, and steroid therapy was resumed. The frequency of diarrhea immediately decreased after starting the second steroid therapy. After discontinuation of steroid therapy, intermittent diarrhea relapsed with abdominal pain. He was diagnosed with obstructive ileus caused by terminal ileum stenosis, and transferred to our hospital for surgical treatment. He did not take any medication regularly before 5-ASA treatment and had no known drug allergy. Physical examination of the head, ears, eyes, nose, and throat, heart, and lungs were within normal limits. His abdomen was soft, mildly distended, and with hyperactive bowel sounds. At the time of admission, his hemoglobin was 9.4 g/dL, and his CRP level was 8.36 mg/dL. The serum total protein level was 4.0 g/dL, and albumin was 1.9 g/dL. His serum IgG4 level was 26 mg/dL (normal range 4.8–105 mg/dL). Small bowel series with endoscopy demonstrated 20 cm of stenosis on the terminal ileum. Contrast-enhanced CT revealed mesenteric fat opacity with pseudocapsule and bowel wall thickening (Fig. 1A and B), which are signs of MP.^[7]^ Abdominal angiography showed no specific findings. Ileal resection was performed and the mesentery was yellow with reddish plaques (Fig. 1C). The pathological findings of the surgical specimen showed marked fibrosis with sclerosis at the mesentery (Fig. 1D and E). Immunostaining showed normal solute carrier organic anion transporter family member 2A1 (SLCO2A1) expression in the small intestinal mucosa of the resected specimen, indicating chronic enteropathy associated with SLCO2A1 gene (CEAS) could be dismissed for this case. Furthermore, familial Mediterranean fever was ruled out because of the absence of *MEFV* mutations. He was diagnosed with MP not UC. One month after surgical treatment, intermittent diarrhea with no pain relapsed again. Colonoscopy showed mucosal edema with erosive change scattered throughout the colon. The PGE MUM level was 152 μg/g Cre. He was prescribed prednisolone and he had an immediate beneficial response. Five months after initiation of steroid therapy, colonoscopy showed improved mucosal edema. The PGE MUM level decreased to 12.3 μg/g Cre. Figure 2 shows his clinical course.

**Figure 3:**
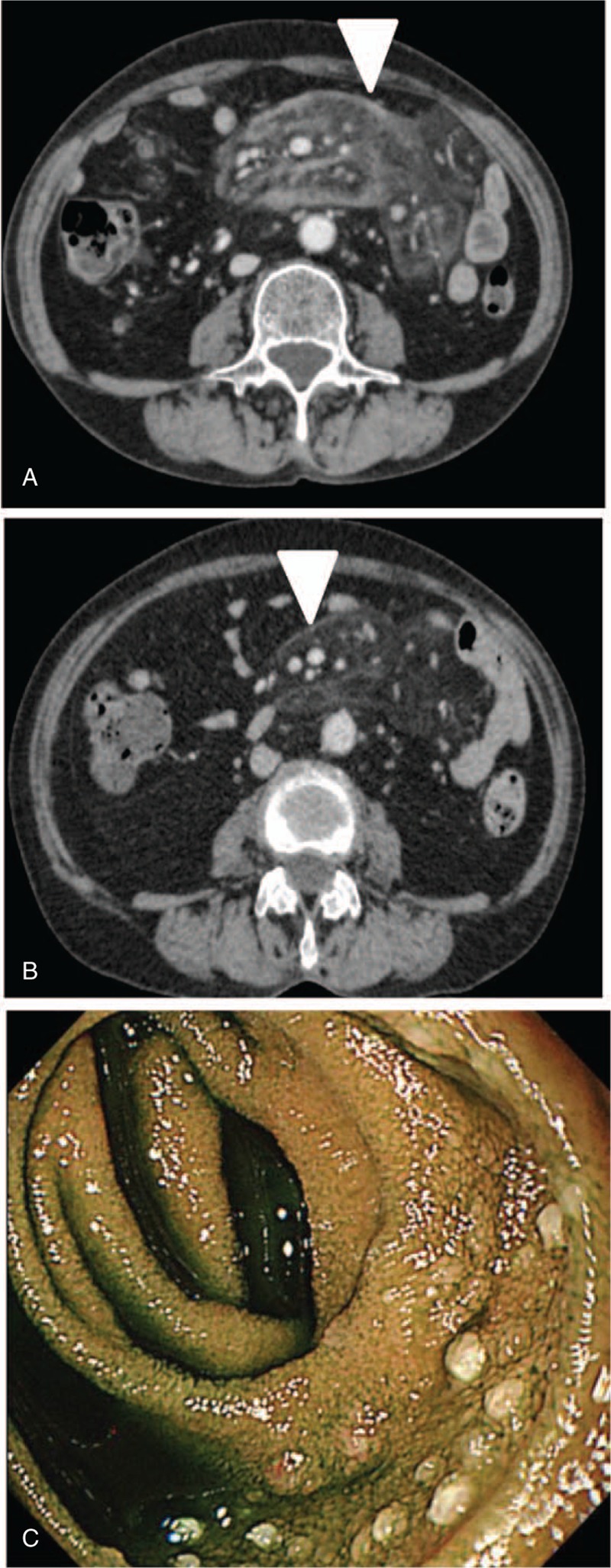
(A) Contrast-enhanced CT performed before steroid therapy showed increased density of mesentery (arrow head). (B) Single-balloon enteroscopy showed white villi in the jejunal mucosa. (C) Contrast-enhanced CT performed after steroid therapy showed improvement of contrast enhancement of mesentery (arrow head).

Case 2: A 67-year-old woman diagnosed with neurofibromatosis type 1 at birth reported abdominal pain, and CT showed contrast enhancement of the mesentery. She was referred to our hospital and diagnosed with MP based on radiological findings without histopathological findings. Her symptom was in remission phase and she was treated with a wait-and-see approach. Three years after diagnosis, abdominal pain relapsed, and CT showed contrast enhancement of the mesentery again (Fig. 3A). Single-balloon enteroscopy showed white granular mucosa in the jejunum (Fig. 3B). Follicular lymphoma stage 1 was confirmed by a positron emission tomography scan, bone marrow aspiration, and pathological examination. Colonoscopy showed no specific findings. Her PGE MUM level was 20.8 μg/g Cre, and the IgG4 level was 8 mg/dL. The lesion site was limited to the jejunum, and she was diagnosed with MP complicated with malignant lymphoma. With respect to lymphoma, routine observation without treatment was recommended by a clinical oncologist of the Division of Hematology. We started steroid therapy for symptomatic MP. Three months after the initiation of steroid therapy, her abdominal pain resolved and CT showed improved contrast enhancement of the mesentery (Fig. 3C). Six months after the initiation of steroid therapy, the PGE MUM level decreased to 15.1 μg/g Cre.

## 3 Discussion

There is no unified diagnostic standard for MP and specific laboratory findings have not been established. In addition to clinical symptoms, contrast enhanced CT and serum markers including CRP have been used for the diagnosis of MP. However, these findings are not specific for MP and previous report showed that MP patient was sometimes misdiagnose with Crohn disease.^[8]^ There is a growing need for pathophysiological and minimally invasive markers for the diagnosis of MP. To our knowledge, this report is the first to suggest PGE-MUM as a potential biomarker for MP.

PGE2 is an inflammatory mediator produced from arachidonic acid via COX, which is then metabolized to stable PGE-MUM. Inflammatory cytokines upregulate the production of COX-2,^[9]^ and this leads to PGE2 secretion in mucosal tissues.^[10]^ High concentrations of PGE2 in inflammatory sites prevents the absorption of electrolytes including sodium and chloride, which stimulates bowel motility.^[11]^ Previous reports demonstrated PGE-MUM levels reflected disease activity in patients with UC^[6]^ and CFIP.^[12]^ Additionally, impaired uptake of PGE led to high concentrations of PGE-MUM in patients with CEAS.^[13]^ Especially in patients with CFIP, reduced expression levels of PGE2 and EP2 receptor in fibroblasts led to perturbed PGE2 signaling, which is involved in fibrosis regulation.^[12]^ Consistent with these reports, our observations support the idea that circumintestinal inflammation with fibrogenesis plays an essential role in the pathology of MP and high PGE-MUM levels. Interestingly, our cases exhibited high PGE-MUM levels regardless of the presence or absence of colonic erosions observed in patients with UC. This suggests that PGE-MUM levels correlate with MP itself but not with colonic inflammation.

PGE-MUM levels in case 1 were much higher than in case 2 before treatment. In addition, the levels sensitively correlated with disease activity in case 1, which had severe fibrosis in the mesentery, during the course of steroid therapy. This finding is similar to a recent report showing PGE-MUM levels in patients with CFIP significantly correlated with fibrosis score.^[12]^ The influence of lymphoma on PGE-MUM levels cannot be completely excluded. The differences in PGE-MUM levels between the 2 cases might be explained by the degree of fibrosis, although histopathological findings of the mesentery were not available for case 2. Cherayil et al^[14]^ characterized 3 histological stages of disease: chronic nonspecific inflammation, fat necrosis, and fibrosis. Cases 1 and 2 might have had markedly different staging.

Taken together, PGE-MUM may be a noninvasive and relatively specific biomarker for MP aiding histological staging without laparotomy biopsy. Furthermore, PGE-MUM contributes to the diagnosis of MP. To verify the usefulness of PGE-MUM, more findings based on clinical studies should be accumulated.

## Acknowledgment

We thank J.L. Croxford, PhD, from Edanz Group for editing a draft of this manuscript.
